# Decision support-tools for early detection of infection in older people (aged> 65 years): a scoping review

**DOI:** 10.1186/s12877-022-03218-w

**Published:** 2022-07-01

**Authors:** Olga Masot, Anna Cox, Freda Mold, Märtha Sund-Levander, Pia Tingström, Geertien Christelle Boersema, Teresa Botigué, Julie Daltrey, Karen Hughes, Christopher B. Mayhorn, Amy Montgomery, Judy Mullan, Nicola Carey

**Affiliations:** 1 Department of Nursing and Physiotherapy, University of Lleida, Lleida, Spain; 2 Health Care Research Group (GRECS), [Lleida Institute for Biomedical Research Dr. Pifarré Foundation], IRBLleida, 25198 Lleida, Spain; 3 School of Health Sciences, University of Surrey, Guildford, GU2 7YH UK; 4 Department of Medical and Health Sciences, Linköping University, Linköping, Sweden; 5 Department of Health Studies, University of South Africa, Pretoria, South Africa; 6 School of Nursing, Faculty of Medical and Health Sciences, University of Auckland, Auckland, New Zealand; 7 Department of Psychology, North Carolina State University, Raleigh, NC 27695-7801 USA; 8 School of Nursing, University of Wollongong, Wollongong, NSW 2522 Australia; 9 School of Medicine, University of Wollongong, Wollongong, NSW 2522 Australia; 10 Department of Nursing and Midwifery, University of the Highlands and Islands, Inverness, IV2 3JH UK

**Keywords:** Decision support tools, Detection, Signs and symptoms, Infection, Older adults

## Abstract

**Background:**

Infection is more frequent, and serious in people aged > 65 as they experience non-specific signs and symptoms delaying diagnosis and prompt treatment. Monitoring signs and symptoms using decision support tools (DST) is one approach that could help improve early detection ensuring timely treatment and effective care.

**Objective:**

To identify and analyse decision support tools available to support detection of infection in older people (> 65 years).

**Methods:**

A scoping review of the literature 2010–2021 following Arksey and O’Malley (2005) framework and PRISMA-ScR guidelines. A search of MEDLINE, Cochrane, EMBASE, PubMed, CINAHL, Scopus and PsycINFO using terms to identify decision support tools for detection of infection in people > 65 years was conducted, supplemented with manual searches.

**Results:**

Seventeen papers, reporting varying stages of development of different DSTs were analysed. DSTs largely focussed on specific types of infection i.e. urine, respiratory, sepsis and were frequently hospital based (*n* = 9) for use by physicians. Four DSTs had been developed in nursing homes and one a care home, two of which explored detection of non- specific infection.

**Conclusions:**

DSTs provide an opportunity to ensure a consistent approach to early detection of infection supporting prompt action and treatment, thus avoiding emergency hospital admissions. A lack of consideration regarding their implementation in practice means that any attempt to create an optimal validated and tested DST for infection detection will be impeded. This absence may ultimately affect the ability of the workforce to provide more effective and timely care, particularly during the current covid-19 pandemic.

**Supplementary Information:**

The online version contains supplementary material available at 10.1186/s12877-022-03218-w.

## Background

Infection is more frequent, and serious in people aged > 65 [1] as they experience non-specific signs and symptoms delaying diagnosis and prompt treatment [2]. Older adults who live in residential aged care are especially vulnerable to infection because of physical and cognitive decline, proximity to other residents and limited resources, as demonstrated during the Covid-19 pandemic [3]. Consequently, nursing home residents experience increased antibiotic usage, clinical complications, hospital admission, and mortality [4, 5].

The terms ‘nursing home’ and ‘residential care’ are defined and used differently between countries [6, 7]. For the sake of comparison, we will in this article use the term ‘nursing home’ to reflect care-homes with on-site qualified nurses; ‘care home’ to reflect those without on-site nursing, and housing that offer access to daily care [7], and ‘residential care’ as an umbrella term combining both, highlighting differences when relevant.

Nursing home residents are > 1.4 times risk of emergency admission and have > 50% unplanned hospital admissions compared to general population aged > 75 years [2, 8]. Unplanned hospital admissions cost the UK National Health Service (NHS) > £11 billion, US healthcare economy >$1.1 trillion/year and account for more than a third of admissions each year, and Swedish healthcare system >SEK 36 trillion a year for people aged > 65 years [9].

It is recognised that guidelines developed by the World Health Organisation can support management and surveillance of infection [10, 11]. However, in order to avoid inappropriate antibiotic therapy, unnecessary hospital admission and risk of complications there is also a need to improve the early detection of infection in older people many of whom present with non-specific clinical manifestations [2, 12–14].

Decision support tools (DST) comprise a wide range of approaches (i.e. algorithms simulation models, and/or techniques and methods) to support the decision making process related to patient care. DST provide a systematic approach to monitoring cognitive and behavioural changes [2, 15, 16] are one approach that could help improve early detection of infection. Ensuring a consistent approach to infection detection, prompt action and treatment [9], DSTs support decision-making and management of the situation [17], and can help reduce unplanned hospital admissions for nursing home residents [18, 19].

Identifying DSTs with the potential to improve detection of infection for older people, particularly for those in residential care is therefore crucial. Given the lack of evidence reporting acceptability and/or feasibility of DSTs for infection detection in older adults [9], a scoping review was undertaken to investigate DSTs designed to support the detection of infection in older people.

## Methodology

A scoping review [20] of evidence published between January 2010–January 2021, and following Preferred Reporting Items for Systematic Reviews and Meta-Analyses extension for Scoping Reviews (PRISMA-ScR) [21]. Scoping reviews are a recognised technique for ‘mapping’ relevant literature, synthesizing and analysing a wide range of research and non-research related material in order to provide greater conceptual clarity about a specific topic or field of evidence. In the present case, the framework adopted was based on Arksey and O’Malley [20], and divided s into five stages.

### Stage 1: identifying the research question

“What DSTs are available to support the detection of infection in older people (aged >65 years)?”

### Stage 2: identifying relevant studies

*Definition of terms:* ‘Decision support tool’ was used as an umbrella term that allowed inclusion of other related concepts such as ‘decision support techniques’, ‘checklists’ or ‘decision aids’ [17].

Searches were conducted during October 2020–January 2021 using MEDLINE, Cochrane, EMBASE, PubMed, CINAHL, Scopus and PsycINFO databases. Database specific index terms, such as MeSH (MEDLINE) were used in searches together with keywords in the title/abstract, and synonyms and wildcard functions used for plurals and differences in US/UK spelling. Boolean logic was used and a string of keyword terms i.e. ‘decision support tool’, ‘clinical assessment tool’, ‘infection’. These sources were supplemented by hand searches. See Appendix 1 for search terms and example search string.

Articles were included that comprised:

- Empirical studies, including meta-analyses, reporting a DST to support detection of suspected infection (including signs, symptoms and bio markers) in people > 65, any setting and by any member of staff.
- DST at any stage of development/implementation to increase understanding regarding the degree of reliability in the diagnosis they can provide (see Appendix 2).
- Peer reviewed articles published in English or Spanish.

Studies were excluded if they reported on DST use in diagnostic testing, predicting mortality, decisions about the use of do not resuscitate, specific therapy/drugs or treatment/procedure (i.e. pre-operative antibiotics), immunizations, or end of life scales. Review papers and grey literature were also excluded.

### Stage 3: study selection

Results (*n* = 6513) were exported into Mendeley and duplicates removed (*n* = 1720) before titles and abstracts were screened in relation to inclusion/exclusion criteria.

**Fig. 1:**
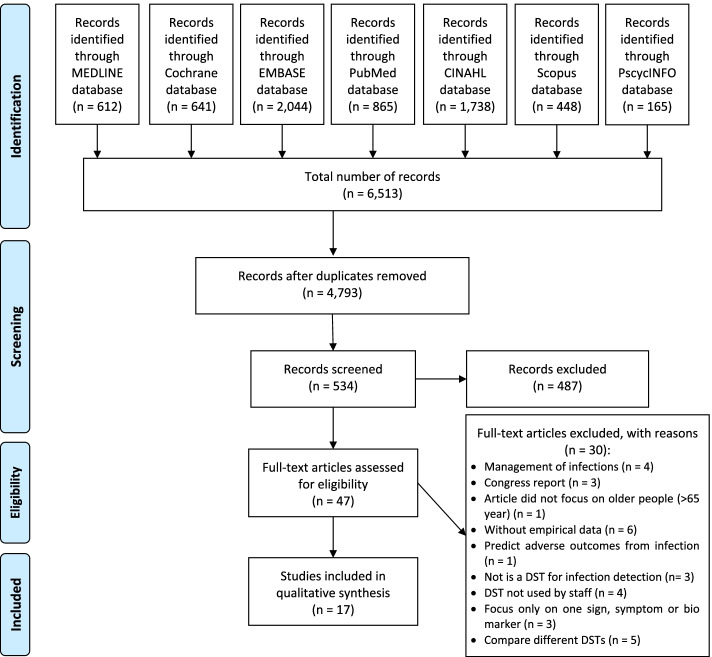
PRISMA Chart

Citations (*n* = 4793) were divided into 10 blocks with each block being independently screened, by title/abstract by two review team members (TB, JD, MS-L, CM, PT, CB, KH, JM, AM, VP). Discrepancies were moderated by four team members (OM, NC, AC, FM). After assessing for eligibility 47 articles were subject to full-text review (OM), with a resultant 17 papers included in the review (Fig. 1).

### Stage 4: data extraction

Data extraction was conducted using a bespoke form to capture details about: study location; study type (methodology); setting and population; DST: stage of development and mode of use; infection(s) type; items or risk factors considered in the DST and results. Extraction was independently completed by one researcher (OM) then reviewed and discussed with three review team members (NC, AC & FM).

### Stage 5: collating, summarising and reporting results

To chart, summarise and synthesise the findings, data extraction forms were used to group the DSTs by stage of development and infection type [16, 22, 23].

## Results

### Characteristics of included studies

Classified by stage of DST development, of the 17 included studies, two reported tool development [24, 25], one development and reliability [26], 7 development and validation [19, 27–32], three validation [33–35] and four testing [36–39] (see Table 1). Based in high income nations, most studies were conducted in single countries including: USA (*n* = 6) [32, 34, 37–39], UK (*n* = 3) [24, 27, 30], Sweden (*n* = 2) with one each from Canada [36], Denmark [35], Germany [33], Japan [26] and Spain [28]. Two international studies reported data from > 1 country [25, 31].

**Table 1:** Overview of included studies

*Author (s), year & country*	*Study type*	*Setting (S) & sample (n)*	*DSTs and its name*	*Mode of use*	*Infection (s) detected*	*Items or risk factors considered in the DST and results*
**Tool development**						
Hughes et al. [24], 2020 UK	Consensus event: i) Literature review, ii) consensus meeting iii) focus groups and interviews	S: Care home: i) consensus meeting (*n* = 4 experts) ii) focus groups (*n* = 6 care home staff & *n* = 6 resident families) and interviews (*n* = 8 GPs)	Algorithm adapted from Loeb et al. [40]	Algorithm	UTI, respiratory tract, skin & soft tissue	One or more new/worsening symptoms: suspected fever, change in behaviour, reduced mobility, loss of appetite and/or the typical infection symptoms.
Van Buul et al. [25], 2018 USA, Netherlands, Canada, Sweden and Australia	Delphi consensus procedure i) Expert panel ii) Delphi rounds ×4	S: Nursing home i) Expert panel (*n* = 15 old care physicians) ii) Response rates to the 4 Delphi questionnaires were 100, 88, 94, and 88%, respectively (same sample as expert panel)	Decision tool for the empiric treatment of suspected UTI in frail older adults	Algorithm	UTI	• No indwelling catheter: recent onset of dysuria, urgency, frequency, incontinence, visible urethral purulence, change in urine colour, macroscopic haematuria, pain, mental status change, general lack of well-being, decreased intake, diarrhoea, nausea, vomiting, malaise, fatigue, weakness, dizziness, syncope, decreased functional status. • Indwelling catheter: no other infectious focus plus at least: fever (> 24 h), rigors/shaking chills, clear-cut delirium (after excluding urinary retention as a possible cause)
**Tool development and reliability**						
Matsusaka et al. [26], 2018 Japan	Retrospective case series review	S: Hospital *n* = 102 bedridden patients receiving oral care	A bedridden patient pneumonia risk (BPPR) score	Checklist	Pneumonia	• Albumin < 3.5 g/dL or/and urine bacteria were the two only risk factors associated independently with pneumonia. Not: age, sex, BMI, WBC, Lymphocyte, CRP, Hb, Iron, TP, TC, BUN, Creatinine, CPK, or Low uric acid. • Total BPPR score is 0,1, or2 (low-moderate and high risk) according to absence or presence of the two risk factors.
**Tool development and validation**						
Rawson et al. [27], 2019 UK	Development & cross validation of supervised machine learning	S: Hospital (*n* = 3) *n* = 104 patients diagnosed with infection within 72 hrs of admission	Supervised machine learning (SML) algorithm for diagnosing bacterial infection	Algorithm	Bacterial	• Microbiology records and six available blood parameters (CRP, WCC, bilirubin, creatinine, ALT and ALP). • Sensitivity and specificity: the infection group had a likelihood of 0.80 (0.09) and the non-infection group 0.50 (0.29) (*P* = 0.01; 95% CI: 0.20–0.40). ROC AUC was 0.84 (95% CI: 0.76–0.91).
García-Tello et al. [28], 2018 Spain	Retrospective cohort study	S: Hospital *n* = 1524 patients with UTI i) development cohort *n* = 1067 (70%) ii) validation cohort: *n* = 457 (30%)	Nomogram to predict the probability of infection by extended-spectrum beta-lactamase (ESBL)-producing microorganisms.	Nomogram model	UTI	• Age, male gender, nursing home residency, previous antimicrobial therapy or hospitalization, recurrent UTI and non-urological invasive procedure. • This nomogram model had a discriminative accuracy of 0.79 (95% CI 0.77–0.82). In the validation cohort, the discriminative accuracy of the model was 0.81 (95% CI 0.77–0.85).
Johansson et al. [29], 2018 Sweden	Development and validation pre-hospital decision support system (DSS)	S: Pre-hospital ED i) Development: *n* = 1921/6323 electronic patient records of adults > 18 yrs. acute infection reviewed; ii) peer review of preliminary DST *n* = 3 clinical experts iii) Evaluation and validation of the DST (theoretical test) 12 cases and 250 nurses, iv) Validation of pre-hospital DSS in prospective pilot study *n* = 72 patients	Pre-hospital DST	Paper based form	Severe respiratory infection, severe central nervous system infection (CNS), and sepsis	• Severe respiratory infection: confusion, respiratory rate ≥ 30/min, SBP < 90 mmHg, sat. O2 < 90%. • CNS infection: fever/chills, and one of: confusion, headache, neck stiffness/back pain, petechiae. • Sepsis: fever + chills, and one of: respiratory rate ≥ 30/min, SBP < 90 mmHg, sat. O2 < 90%. • All required a previous clinical suspicion. • Validation cohort: the positive predictive value was 94% (32/34 cases) and for 30 of the 34 patients (88%).
Siaw-Sakyi [30] 2017 UK	Development: consensus event: comprising audit & expert panel Validation: Audit pre and post use of the WIRE tool	S: Community Development: i) Audit of 1500 patient ii) Expert panel of tissue viability nurses: series of group meetings, Validation: *n* = 55 patients, 48 wounds. Analysis based on 150 WIRE scores and 47 swab results	Wound Infection Risk- Assessment and Evaluation tool (WIRE)	Checklist	Wound infection	• Pain; slough/necrotic tissues; friable/unhealthy granulation, bed colour; exposure of underlying organs; pocketing/ tunnelling/bridging; non-healing/wound breakdown; maceration/ excoriation; erythema; localised heat; swelling/oedema; crepitus; wound size and depth; type of wound; exudate; diabetes; immunosuppression/ cytotoxic/chemotherapy; cardiac/circulatory; malnutrition; smoking; non-steroidal anti-inflammatory drugs; steroids; multiple antibiotic therapy; lack of concordance, multiple hospital admissions; recurrent wound infections; environmental factors; temperature; pulse rate; blood pressure; respiratory rate, altered mental ability; rigors; nausea/vomiting; and lymphangitis • 117/150 (78%) cases were matched between the swab & WIRE result confirming presence of infection.
Tingström et al. [19], 2015 Sweden	Development & validation	S: NHs (*n* = 6) *n* = 204 nursing home residents over 1 year Development: multi-stage 2006–2014 resulting in 13 item tool; 388 infection events	Clinical decision-making process. Early Detection of Infection Scale (EDIS) instrument [41].	Algorithm	All type of infections	• Items of EDIS: discomfort, unrestrained, aggressiveness, restlessness, confusion, infirm, decreased eating, pain, general signs and symptoms of illness (for example fever, shaking, etc.), Respiratory symptoms, UTI symptoms, Wound infection symptoms and abnormal breath per minute. • Content validity analysis: 12/13 of the items correlated significantly with at least one other statement. • Construct validity: “temperature”, “respiratory symptoms” and “general signs and symptoms of illness” were significantly related to “infection”. These last items predicted correct alternative responses in 61% of the cases.
Afonso et al. [31], 2012 USA and Switzerland	Development & validation clinical decision rule	S: USA: hospital (*n* = 258), ED & Switzerland: primary care (*n* = 201) Secondary analysis of two combined existing data sets Development set *n* = 322 patients (70%) Validation set *n* = 137 patients (30%)	Decision tree for the diagnosis of influenza	Classification and regression tree	Influenza	• Of the three models: regression reliability and validated, model 2 presented best results and classified two-thirds of patients as low or high risk and had an area under the receiver-operating characteristics curve (AUROCC) of 0.76. • Patient with suspected influenza have > 37 °C: high risk of flu (58%). And if they do not have fever, but do have chills and/or sweating, flu risk was 18%.
Chumbler et al. [32], 2010 USA	Development and validation of clinical prediction rule	S: Hospitals (*n* = 5) Secondary analysis retrospective cohort study (total *n* = 1363) Development set *n* = 925 patients (70%). Validation group *n* = 438 patients (30%)	Post-stroke pneumonia prediction system	Logistic regression model	Post-stroke pneumonia	• Abnormal swallowing & history of pneumonia (4 points); followed by greater NIHSS score (3 points); patient being ‘found down’ at symptom onset (3 points); and age > 70 years (2 points). • The discriminatory accuracy of the 3-level clinical prediction rule denoted low, medium and high risks of pneumonia. This exceeded the acceptable range in both the development group (c statistic: 0.78) and validation group (c statistic: 0.76).
**Tool validation**						
Gräff et al. [33], 2017 Germany	Retrospective observational study	S: ED *n* = 20,836 patients > 16 years	Manchester Triage System (MTS) Adaptation	A computer algorithm	Sepsis	• Breathlessness; heart rate: > 120; Temperature (°C): < 35 or > 41 (orange)/ > 38.5 (yellow); Blood pressure: only mention for pregnancy. • MTS triage categories of ‘yellow’, ‘orange’ or ‘red’. MTS category ‘green’ or ‘blue’ was judged to be inadequate prioritisations. Patients with severe sepsis with circulation dysfunction were considered adequately categorised only when allocated to ‘orange’ or ‘red’. • Patients with severe sepsis were appropriately prioritised with a sensitivity of 84.5% (95% CI 78.1 to 89.4), and LR– was 0.330 (95% CI 0.243 to 0.450). In the group with severe sepsis and circulation dysfunction, sensitivity was 61.5% (95% CI 39.3 to 79.8), and LR– was 0.466 (95% CI 0.286 to 0.757).
Walchok et al. [34], 2017 USA	Retrospective case series review	S: Mixed rural and suburban community 946/1154 patients with sepsis alert and blood culture	Pre-hospital Sepsis Assessment Tool (Pre-SAT) [42]	Paper form	Sepsis	• SIRS criteria [43] + mechanical ventilation, and/or signs of poor perfusion (systolic blood pressure < 90 mmHg). • 2 signs of SIRS and a known or suspected source of infection required the paramedic to issue a ‘Sepsis Alert’ to the receiving ED. These criteria were used after gaining consensus from the two receiving hospital medical sepsis committees. • 848/1154 confirmed overall sepsis diagnosis: Positive blood culture 179/946 (18.9%). Antibiotics administered in 72/100 patients
Jessen et al. [35], 2016 Denmark	Retrospective matched cohort study	S: ED *n* = 420 Bacteremia (*n* = 105) Non-bacteremia (*n* = 315)	Clinical decision rule to predict Bacteremia in the ED [44]	Clinical decision rule	Bacteremia	• Suspected endocarditis (3 points); temperature > 39.4 °C (103.0 °F) (3 points); indwelling vascular catheter (2 points); and minor criteria (1 point each): age > 65 years, temperature 38.3–39.3 °C, chills, vomiting, hypotension (SBP < 90 mmHg), white blood cell count > 18,000 cells × 109/l, bands > 5%, platelets< 150,000 cells × 109/l and creatinine> 177 μmol/l (2.0 mg/dl). • The sensitivity of the prediction rule was 94% (95% CI, 88–98%) and the specificity was 48% (95% CI, 42–53%). The AUROCC was 0.83.
**Tool testing**						
Pasay et al. [36], 2019 Canada	Cluster RCT of	S: Rural NHs (*n* = 42), *n* = 21 allocated to each group *n* = 1001 residents	The UTI in LTC Facilities Checklist	A clinical decision-making tool without laboratory test	UTI	• No indwelling catheter: Acute dysuria or Temp > 38 °C or 1.1°above baseline on 2 consecutive occasions (4–6 hr. apart). Plus: increased urinary frequency, urgency, incontinence, flank or suprapubic pain or tenderness, and haematuria. • Indwelling catheter: No other cause of infection and ≥ 1 of: Temp > 38 °C or 1.1°above baseline on 2 consecutive occasions (4–6 hr. apart), flank or suprapubic pain or tenderness, rigors and delirium. • UTI symptoms were charted in 16% of cases and that urine culture testing occurred in 64.5% of cases (regression coefficient, *p* = 0.02; 95% CI, 0.001–0.03). Significantly decreased the rate of urine culture testing and antimicrobial prescriptions for UTI (*p* < 0.001), with no increase in hospital admissions or mortality.
Amland & Hahn-Cover [37], 2016 USA	Retrospective cohort study	S: Medical centres (a level 1 trauma centre, a level 2 trauma centre, a women’s and children’s hospital, and 2 community hospitals). *n* = 6200 patients aged over 65 years	Clinical decision support system (CDS) based on SIRS [43]	Cloud-based computerized system	Sepsis	• ≥3 of the following 5 criteria were satisfied: (1) temperature > 38.3 °C or < 36 °C; (2) heart rate > 95 beats/min; (3) respiratory rate > 22 breaths/min; (4) white blood cell count > 12,000 cells/mm3 or < 4000 cells/mm3, or > 10% immature (band) forms; or (5) glucose 141 to < 200 mg/dL. • ≥2 criteria were present and ≥ 1 of the following 4 organ system dysfunction criteria were satisfied: (1) cardiovascular system, SBP < 90 mmHg and/or mean arterial pressure < 65 mmHg; (2) tissue perfusion, serum lactate > 2.0 mmol/L; (3) hepatic system, total bilirubin ≥2.0 mg/ dL and < 10.0 mg/dL; and (4) renal system, serum creatinine ↑0.5 mg/dL from baseline. • 83% sensitivity and 92% specificity.
McMaughan et al. [38], 2016 USA	RCT	*n* = NHs (*n* = 12) 699 prescriptions for suspected UTI for *n* = 547 NH residents	Decision-making aid for suspected UTI.	Paper form	UTI	• Acute dysuria; fever (> 37.9 °C) or 1.5 °C increase above baseline temperature; new or worsening urgency, frequency, or incontinence, suprapubic pain; gross haematuria; costovertebral angle (flank) tenderness; rigors, and delirium (recent and abrupt change in mental status). • The odds of a prescription decreased significantly in homes that succeeded in implementing the decision-making aid (OR = 0.35, 95% CI = 0.16–0.76), compared to homes with no fidelity.
Umberger et al. [39], 2016 USA	Secondary analysis of a retrospective case series review	*n* = Hospital, ICU *n* = 77 patients with sepsis	Candida Score [45, 46]	Paper form	Candidemia	• Severe sepsis (2 points), surgery at baseline (1 point), total parenteral nutrition (1 point), and Candida colonization (1 point). • Infection detection with score ≥ 3 points. • Sensitivity was 50%, specificity was 68.1%, positive predictive value was 15.4%, and negative predictive value was 92.2%.

Most hospital based DSTs were designed for use only by physicians [31, 32, 35, 37, 39]. DSTs developed in NHs or care homes adopted a more multi-professional approach for use by physicians [24, 25, 36], nurses [24, 36, 38] and/ or nurse assistants [19, 24, 36]. The involvement of healthcare staff, using DSTs were also applicable to community care [30, 31, 34] and pre-hospital care [29].

### Setting

Nine studies were based in the hospital [26–28, 31–33, 35, 37, 39], including two in an emergency department (ED) [33, 35] and one intensive care unit (ICU) [39]; four in nursing homes (NHs) [19, 25, 36, 38]; three in community [30, 31, 34], including one community ED [31]. One study was based in a pre-hospital ED (ambulance services) [29] with only one based in a care home [24].

### Infection type

Fourteen articles focused on detecting a single type of infection including: pneumonia [26, 32], influenza [31], bacteraemia [27, 35], urinary tract infection (UTI) [28], sepsis [33, 37] and candida [39] in the hospital; UTI in nursing homes [25, 36, 38]; and wound [30], influenza [31] and sepsis [34] in the community. A tool to detect severe respiratory and central nervous system infection, and sepsis was developed for use in pre-hospital services [29]. Only two studies based in a nursing or care home reported on a tool to detect non-specific infection [19, 24].

A heterogeneous range of signs and symptoms were used to inform DST content. A summary of the signs and symptoms associated with the three most common types of infection addressed by DSTs i.e., sepsis, UTI and respiratory tract infection can be found in Table 2.

**Table 2:** Signs and symptoms and other factors included in Decision Support Tools

**1. Sepsis**																						
	Fever	Temperature (°C): < 36	Breathlessness	Sat. O2 < 90%	PaCO2 < 32 mmHg	Mechanical ventilation	SBP < 90 mmHg	Mean arterial pressure < 65 mmHg	Raised heart rate	WBC > 12,000/μL or < 4000/μL	Glucose 141 to < 200 mg/dL	Tissue perfusion: lactate > 2.0 mmol/L	Altered hepatic system	Altered renal system	Known or suspected source of infection	**Total number** ***n*=**						
Johansson et al. [29], 2018	✓	✓	✓	✓			✓									**5**						
Gräff et al. [33], 2017	✓	✓	✓						✓							**4**						
Walchok et al. [34], 2017	✓	✓	✓		✓	✓	✓		✓	✓					✓	**9**						
Amland & Hahn-Cover [37], 2016	✓	✓	✓				✓	✓	✓	✓	✓	✓	✓	✓		**11**						
**Total** ***n*=**	**4**	**4**	**4**	**1**	**1**	**1**	**3**	**1**	**3**	**2**	**1**	**1**	**1**	**1**	**1**							
**2. Urinary Tract Infection**																						
	Catheter	Acute dysuria	Flank or suprapubic pain	Haematuria	Fever	Urinary urgency/frequency	Urinary incontinence	Mental status change (Delirium)	Rigors	Age	Male	Living in nursing home	Previous antimicrobial therapy	Previous hospitalization	Recurrent UTI	Non-urological invasive procedure	Urethral purulence	Change in urine colour	Decreased intake	Gastrointestinal symptoms	Functional status decrease	**Total number** ***n*=**
Pasay et al. [36], 2019	No-catheter	✓	✓	✓	✓	✓	✓														✓	**7**
	Catheter		✓		✓			✓	✓													**4**
García-Tello et al. [28], 2018										✓	✓	✓	✓	✓	✓	✓						**7**
Van Buul et al. [25], 2018	No-catheter	✓	✓	✓		✓	✓	✓									✓	✓	✓	✓	✓	**11**
	Catheter				✓			✓	✓													**3**
McMaughan et al. [38], 2016		✓	✓	✓	✓	✓	✓	✓	✓												✓	**9**
**Total** ***n*=**		**3**	**4**	**3**	**4**	**3**	**3**	**4**	**3**	**1**	**1**	**1**	**1**	**1**	**1**	**1**	**1**	**1**	**1**	**1**	**3**	
**3. Respiratory Tract Infection**																						
	Type of infection	Abnormal swallowing result	Age > 70 years	Albumin < 3,5 g/dL	Chills or sweating	Mental status change (Delirium)	History of pneumonia	NIHSS score	Respiratory rate ≥ 30/min	Sat. O2 < 90%	SBP < 90 mmHg	Temperature > 37 °C	Urine bacteria	**Total number** ***n*=**								
Johansson et al. [29], 2018	Severe respiratory infection					✓			✓	✓	✓			**4**								
Matsusaka et al. [26], 2018	Pneumonia			✓									✓	**2**								
Afonso et al. [31], 2012	Influenza				✓							✓		**2**								
Chumbler et al. [32], 2010	Post-stroke pneumonia	✓	✓			✓	✓	✓						**5**								
**Total** ***n*=**	**1**	**1**	**1**	**1**	**2**	**1**	**1**	**1**	**1**	**1**	**1**	**1**										

Fever and breathlessness were included as signs and symptoms reported in all four sepsis DSTs [29, 33, 34, 37]. Other signs and symptoms i.e. temperature < 36 °C and increased heart rate were included in three of the four sepsis tools, as they are also recognised criteria used to identify Systemic Inflammatory Response Syndrome (SIRS) [43]. A variety of signs and symptom (range *n* = 3–11) including acute dysuria, flank or suprapubic pain, haematuria, fever, urinary urgency/frequency, incontinence, and mental status change [25, 28, 36, 38] were frequently considered in DSTs designed to detect UTI. Of the four DSTs designed to detect respiratory tract infection [26, 29, 31, 32], change in mental status was the only sign or symptom considered in more than one DST [29, 32]. Other signs including oxygen saturation < 90%; fever, UTI, or aged > 70 years old were also considered by some authors [26, 29, 31, 32].

### Stage of DST development and applications

DSTs are presented by their stage of development i.e. ‘*development’*; ‘*development and reliability’*, ‘*development and validation’*, ‘*validation’ and ‘testing*‘to increase understanding regarding their readiness for implementation and potential adoption in clinical practice in residential care [15, 16, 22, 23].

#### Development

Articles reporting DSTs at the ‘*development stage’* (*n* = 2) each outlined an algorithm to improve infection detection: one focused on UTI [25], whereas Hughes et al. [24] considered three common infections (UTI, respiratory tract infection, skin and soft tissue infection).

Using a Delphi panel and series of consensus events Van Buul et al. [25] identified that UTI detection in frail older adults living in nursing homes was affected by the presence of a urinary catheter. A wide range of symptoms i.e., urinary, gastrointestinal, mental status change, general lack of well-being and decreased functional status for example, needed to be considered in those without catheters (see Table 2). For those with a catheter however, the algorithm required presence/absence of fever (> 24 h), rigors/shaking chills and clear-cut delirium in order to detect a UTI.

More recently, an algorithm based DST for use with common infections in UK care home residents was developed by Hughes et al. [24]. Informed by the Canadian-based Loeb et al. criteria [40] a multi-faceted approach including literature review, consensus meeting, focus groups and interviews was adopted to help improve management of the three infections (UTI, respiratory tract infection, skin and soft tissue infection) [24]. Based on presence of fever, change in functional status and psychological behaviour a revised and adapted algorithm describing management in terms of initial assessment, observation and action by the care home staff was produced (see Table 1).

#### Development and reliability

One study reported on the ‘*development and reliability’* of a DST to detect pneumonia in hospital based patients, the ‘Bedridden Patient Pneumonia Risk’ (BPPR) [26]. Analysis of multiple risk factors confirmed that albumin < 3.5 g/dL and/or urinary bacteria were the only two risk factors associated independently with pneumonia. The resultant BPPR therefore is based on a score of 0, 1 or 2 according to their absence or presence (see Table 1).

#### Development and validation

Seven studies reported ‘*development and validation’* of DSTs, five focused on specific infections [27–29, 31, 32], including three respiratory [29, 31, 32]; one wound infections [30] and one general [19] (see Tables 1 & 2).

Analysing data from a cohort of hospital based stroke patients Chumbler et al. [32] used logistic regression to inform the post-stroke pneumonia prediction system. Of the 22 variables considered in the development process, only dysphagia, history of pneumonia, National Institute of Health Stroke Scale (NIHSS) score, decreased cognitive and functional capacity and age > 70 years were independently associated with pneumonia. The discriminatory accuracy of the 3-level clinical prediction rule denoted low-risk (0 points; no risk factors present), medium-risk (presence of 1–3 risk factors) and high risks of pneumonia (4 or more risk factors). Authors [32] concluded that this clinical scoring system may be particularly relevant for hospitals using information technology systems.

Three exploratory models were used by Afonso et al. [31] to develop and validate a decision tree for the diagnosis of influenza with three models in the ED and primary care. Model 1 comprised seven terminal nodes based on temperature, symptom onset, presence of chills, cough and myalgia, whereas a simple tree with only two splits based on temperature and presence of chills was used for Model 2. Similarly model 3 had only two splits based on presence of fever and myalgia, with temperature treated as dichotomous variable (> 38 °C). Model 2 emerged as the most reliable model correctly classifying two thirds of patients as either low or high risk and in need of further evaluation for influenza, and treatment.

Finally, Johansson et al. [29] developed and validated a pre-hospital DST for detecting severe respiratory infection. With a required previous clinical suspicion, respiratory tract infection was detected by delirium, respiratory rate ≥ 30/min, systolic blood pressure < 90 mmHg, oxygen saturation < 90%. This pre-hospital DST however, was validated for diagnosis of severe central nervous system infection, and sepsis (Tables 1 & 2).

Two studies focussed on bacterial infection [27] and UTI [28], respectively. Rawson et al. developed a supervised machine learning (SML) algorithm for diagnosing any hospital based bacterial infection. In this case, microbiology records and six available blood parameters (C-reactive protein (CRP), white cell count (WCC), bilirubin, creatinine, alanine aminotransferase (ALT) and alkaline phosphatase) were used to detect bacteriemia. The validity results showed that those with infection were older and had a greater median CRP; WCC and ALT.

To predict probability of UTI by extended-spectrum beta-lactamase (ESBL)-producing microorganisms, García-Tello et al. [28] developed and validated a nomogram, a two-dimensional diagram designed to allow the approximate graphical computation of a mathematical function. Seven variables including sociodemographic data, history of UTI and living in a nursing home, were considered with results confirming the nomogram had reasonable accuracy in predicting the risk of infection by ESBL-producing bacteria (see Table 1).

Aiming to improve the detection of wound infection, Siaw-Saky [30] developed a ‘Wound Infection Risk- Assessment and Evaluation tool’ (WIRE) comprising three categories medical history (i.e. diabetes or malnutrition), local signs and symptoms (i.e., pain or erythema), and systemic signs and symptoms (i.e., temperature and rigors). Using audit data, the presence of infection was confirmed in 117/ 150 (78%) cases whose wounds were subject to both WIRE and swab assessment.

Finally, a clinical decision-making algorithm, ‘Early Detection of Infection Scale ‘EDIS, for detecting all type of infections in older adults living in NHs was developed by Sund-Levander [41] and validated by Tingström et al*.* [19] for use by Swedish care workers. Validation of the 13 item EDIS tool suggested that ‘he/she is not as usual’ along with ‘increased temperature’, and presence of ‘respiratory symptoms’ and/or ‘general signs and symptoms of illness’ made by nursing assistants should be taken seriously, and lead to follow up by a nurse or physician.

#### Validation

Three studies [33–35] reported on DST ‘*validation*’. Two studies focused on detecting sepsis [33, 34] were based on the signs and symptoms of SIRS [43] (see Tables 1 & 2); and one bacteriemia [35].

Using a computer algorithm, and categorising urgency of ED patients into ‘immediate’, or ‘within 10 or 30 minutes’ the ‘Manchester Triage System’ (MTS) [33] was validated for use as a ‘sepsis alert’, with results indicating the that the tool had significant potential to improve prioritisation and treatment of ED patients with septic illness.

Similarly, Walchok et al. [34] validated the ‘Prehospital Sepsis Assessment Tool’ (Pre-SAT) [42] (see Tables 1 & 2). The criteria were used after gaining consensus from the two receiving hospital systems EMS sepsis committees were that having two signs of SIRS and a known or ‘suspected source of infection’ required the paramedic to issue a ‘sepsis alert’ to the receiving ED. In terms of effectiveness, the application of this criteria provoked that EMS administered antibiotics matched blood culture growth in 72% of patients.

In order to improve clinical guidance regarding the need for obtaining blood cultures, Jessen et al. [35] validated a clinical decision rule to support rapid bedside estimation of bacteremia risk. Using several signs and symptoms i.e. suspected endocarditis; temperature; indwelling vascular catheter; age > 65 years; chills; vomiting; hypotension; white blood cell count; bands; platelets; and creatinine this DST was developed to support ED physicians and treatment consensus.

#### Testing

Testing of DSTs, to confirm factor structure on an independent data set, was used to determine how well the measured variables represent the number of constructs in four studies [36–39]: two UTI [36, 38], one sepsis [37] and one candidemia [39].

Both studies exploring DSTs for UTIs were undertaken in nursing homes and used the same signs and symptoms (see Table 2). Interesting, the ‘UTI Long-term care (LTC) Facilities Checklist’ [36] detects infection by considering whether or not residents are catheterised, whereas the decision-making aid for suspected UTI tested by McMaughan et al. [38] does not make this differentiation (see Tables 1 & 2).

Using a clinical decision support system (CDS) for early recognition of sepsis, the DST tested by Amland and Hahn-Cover [37] included SIRS criteria [43], cardiovascular items and blood analytical parameters (see Tables 1 & 2). The authors report the system’s activation rate appears to be acceptable in terms of being consistent with a flow sheet paradigm for capturing results, clinical events, and time stamps, These indicate [37] that future quality improvement initiatives should include the application of the sepsis CDS across patient care processes.

Finally, candida scores [45, 46] in ICU patients with sepsis were used to test a 4 item DST for candidema by Umberger et al. [39]. Infection was confirmed with a score of ≥3 points and based on items related to severe sepsis (2 points), surgery at baseline (1 point), total parenteral nutrition (1 point), and Candida colonization (1 point). Despite a relatively poor sensitivity (see Table 1), Umberger et al. [39] results indicate a reasonable specificity with a strong negative predictive value proposing this make this tool a viable option for screening medically ill patients who may require antifungal agents.

## Discussion

This scoping review found a diverse group of DSTs available to support detection of infection in older people at varying stages of development, largely focused on specific types of infection, with few based in the nursing or care home setting (*n* = 5) [19, 24, 25, 36, 38]. Each article reported a different DST and only two reported a DST based on the adaptation of a previously developed tool [24, 33]. In addition to wider concerns regarding deficits in knowledge utilisation, and the need to ensure more efficient use of resources, the heterogenous nature of the DST dataset for infection detection reflects the need to expedite the translation of research findings in to clinical practice [47]. Given the concerns regarding the projected rise in older people, subsequent increase in the number of nursing and care home residents [13, 14, 48] and impact on future service utilisation, this review is timely and of international relevance. It is the first of its type to chart and synthesize the evidence on this issue.

Our review found DSTs for improving infection detection in a broad range of settings in high income countries. However, results highlighted that most infection detection DSTs had been developed for use in hospitals [26–28, 31–33, 35, 37, 39], in single countries [24, 26, 36–39, 27–30, 32–35]. We found no evidence exploring the feasibility of using these tools in other settings and/ or by other groups of health and social care professionals. While it is important to acknowledge the majority of articles reported key stages of DST tool development and testing, the lack of attention given to any aspect of implementation, feasibility and or acceptability in practice is significant.

The scope of the services, funding and legislative requirements vary considerably between countries and care settings [7]. Early consideration of implementation, as outlined by the MRC complex intervention framework [49], is important to ensure adoption and implementation at scale. Paying attention to cultural and contextual differences along with factors that facilitate, or hinder implementation is therefore key to ensuring the benefits of innovation in practice such as DSTs to help improve early detection of infection are fully realised.

Reviewed DSTs largely focused on single types of infection, and most did not specifically explore their use solely in older people [26–28, 31–34, 37, 39]. Although it is established that older people experience common infections including UTI, respiratory tract infection and wound infections more frequently [50], physiological changes associated with aging, and chronic diseases such as diabetes, dementia and stroke [41] mean they often exhibit non-specific signs and symptoms delaying diagnosis and treatment, particularly those in residential care [51, 52]. Consequently, DSTs that have been designed for a specific infection have limited applicability in residential care settings. Additionally, organisational factors such as staff ratios, workload, lack of specialist knowledge, and variable training of nursing assistants and carers [7] means it would not be practical to use multiple DSTs to detect the various types of infection experienced by nursing and care home residents. There is therefore a need for DSTs that detect infection in general for older adults who live in residential care. For example, the Early Detection of Infection Scale (EDIS), one of only two general infection DSTs included in this review [19, 24] is designed for completion by Swedish care workers who ultimately have the most direct contact with residents and tend to be the first people to identify change in psychological and or cognitive behaviour [41, 53].

Our results indicate that the use of DSTs for infection detection in older people is an emergent area of practice, with most studies reporting tool development and testing. Of the four studies reporting DST testing [36–39], two were based in nursing homes [36, 38], with only one designed for use by nurses, physicians and care workers [36]. The lack of robust evidence regarding the benefits or otherwise of DSTs in this area means that results should be treated with a degree of caution while the included tools are subject to further investigation.

In the UK alone, the National Patient Safety Agency National Reporting & Learning System report 7% of deaths/severe harm incidents in general are related to unrecognised infection [54]. Robust tools are used in acute care settings for early identification of deterioration, e.g. National Early Warning Score (NEWS), but as this review has shown are not commonly developed for use in residential care or by those providing the bulk of care in this setting i.e. nurses and care workers.

In addition to reducing spread of covid-19, evidence suggests training nursing and care home staff to recognise and communicate signs of deterioration through DSTs can provide patient benefit by reducing and/or preventing hospital transfers [53, 55–57]. Having improved instructions about what to do next was reported to be the most important action to help improve care of residents with suspected infection by 40% of 204 nurses and care workers recently surveyed in England, Sweden and Spain [58]. While nearly 90% reported they used DSTs for pressure sores, falls and pain, < 50% were aware of use for detecting infection. There is therefore a need for a step-change in how DSTs for infection detection in older people in residential care are developed and implemented in practice.

### Limitations

We conducted a comprehensive search using key databases and hand searches. It is possible however that some papers may have been missed. Only papers in English and Spanish were included which means there could be other relevant papers. Grey literature was excluded, and hence it is also possible we could have missed evidence on DSTs that are already used in practice.

## Conclusions

This scoping review has explored DSTs available to support detection of infection in older people. DSTs provide an opportunity to ensure a consistent approach to the early detection of infection supporting prompt action and treatment, thus avoiding emergency hospital admissions. The small number of DSTs that have undergone testing in residential care suggests a significant gap in the literature. Relatedly, given that older people often exhibit non-specific signs and symptoms, it was surprising that only two eligible studies reported DSTs to detect infection in general. Despite this, the results suggest that DSTs for infection detection are being used for a broad range of infections, and different settings. However, until consideration is given to their implementation in practice, any attempt to create an optimal validated and tested DST for infection detection will be impeded. This absence may ultimately affect the ability of the workforce to provide more effective and timely care, particularly during the current covid-19 pandemic.

## Supplementary Information

**Additional file 1.**

**Additional file 2.**

## Data Availability

Not applicable.

## Abbreviations

ALP: Alkaline Phosphatase
ALT: Alanine Aminotransferase
AUC: Area under the curve
AUROCC: Area under the receiver-operating characteristics curve
BUN: Blood Urea Nitrogen
CI: Confidence interval
CNS: Central nervous system
CPK: Creatinine Phosphokinase
CRP: C-Reactive Protein
ED: Emergency Department
Hb: Haemoglobin
LR: Likelihood ratio
LTC: Long Term Condition
NIHSS: National Institute of Health Stroke Scale
MRC: Medical Research Council
ROC: Receiver Operating Characteristic
SAT: Saturation
SBP: Systolic Blood Pressure
SIRS: Systemic Inflammatory Response Syndrome
TC: Total Count
TP: Total Protein
USA: United States of Americas
UTI: Urinary tract infection
WCC: White cell count
