# Supplementary material for: Decision support-tools for early detection of infection in older people (aged> 65 years): a scoping review
Source: BMC Geriatr. 2022 Jul 1;22:552. doi: 10.1186/s12877-022-03218-w (PMC9247966; doi:10.1186/s12877-022-03218-w)
Supplement: Supplementary file 1 — Additional file 1. [file 12877_2022_3218_MOESM1_ESM.docx]

**Appendix 1. Search terms and example of search string**

**Search terms**

Searches were conducted using Medline, Cochrane, EMBASE, PubMed, CINAHL, Scopus and PsycINFO databases, via the EBSCO platform. Database specific index terms, such as MeSH (MEDLINE) were used in searches together with keywords in the title/abstract, and synonyms and wildcard functions used for plurals and differences in US/UK spelling. Boolean logic was used. Searches were based on the following terms: "Decision Support Systems, Management", “Decision Theory", "Decision Making", “Decision Support Systems, Clinical", "Decision support tool*", "Decision support tool*", "Clinical Decision-Making", "Decision Support Techniques", "Checklist", "Decision tool*", "Decision aid*", "Clinical Assessment Tool*", "Infection*", "Skin Diseases, Infectious", "Wound Infection", "Common Cold", “Rhinovirus", “Influenza, Human", “Chest infection*", "Pneumonia", "Respiratory Tract Infections" and "Urinary Tract Infections".

**Example of search string**

| Platform: EBSCO | |
| --- | --- |
| Database: **MEDLINE** | |
| Limits: 2010 –2020; Human; English and Spanish; Aged 65+ years | |
| # Hits: 612 |  |
| 1 | (MH "Decision Support Systems, Management") |
| 2 | (MH "Decision Theory") |
| 3 | (MH "Decision Making") |
| 4 | (MH "Decision Support Systems, Clinical") |
| 5 | TI "Decision support tool*" |
| 6 | AB "Decision support tool*" |
| 7 | "Clinical Decision-Making" |
| 8 | (MH "Decision Support Techniques") |
| 9 | "Checklist" |
| 10 | "Decision tool*" |
| 11 | "Decision aid*" |
| 12 | "Clinical Assessment Tool*" |
| 13 | OR/1-12 |
| 14 | (MH "Infection*") |
| 15 | TI "Infection*" |
| 16 | AB "Infection*" |
| 17 | (MH "Skin Diseases, Infectious") |
| 18 | (MH "Wound Infection") |
| 19 | (MH "Common Cold") |
| 20 | (MH "Rhinovirus") |
| 21 | (MH "Influenza, Human") |
| 22 | "Chest infection*" |
| 23 | (MH "Pneumonia") |
| 24 | (MH "Respiratory Tract Infections") |
| 25 | (MH "Urinary Tract Infections") |
| 26 | OR/14-25 |
| 27 | 13 AND 26 |
| **Key** | |
| Ti– title word | |
| Ab – abstract word | |
| MH – Main index/ MeSH term | |
|  |  |
