# Supplementary material for: Decision support-tools for early detection of infection in older people (aged> 65 years): a scoping review
Source: BMC Geriatr. 2022 Jul 1;22:552. doi: 10.1186/s12877-022-03218-w (PMC9247966; doi:10.1186/s12877-022-03218-w)
Supplement: Supplementary file 2 — Additional file 2. [file 12877_2022_3218_MOESM2_ESM.docx]

**Appendix 2. The different stages of tool development**

The approach on which the tool development described in this manuscript is based follows the recommendations of Kumar [1]. The author reviews tool development procedures published in leading journals from 1992 to 2007. First, item generation has to base in theoretical evidence. Next step is reliability in that internal consistency, equivalence by inter-rater, and stability by test-retest method. The validity is calculated by testing the content, concurrent and construct. Then, Altman and Bland [2,3] defined the final step of a tool development. The final questionnaire was administered to an independent sample calculating the psychometric characteristics of the tool (i.e. sensivity, specificity or their relationship, the area under the receiver operating characteristic curves (AUROCC)) (see following table). With this in mind, it has been necessary to analyse the development status of each Decision Support Tool included in this review in order to reflect on its possible implementation and adoption in clinical practice of the residential care settings. The deployment of new tools is necessary to standardize care. Providing reliable and validated tools to health professionals is key to increasing the quality of care provided in residential care settings [1,4].

| Stages | Process |
| --- | --- |
| Tool development [1,4] | - Researcher examine the extant theory.  - The items from a theory of the construct (latent variable) should be developed  - Once the researcher has generated the initial pool of items, the next step involves having a panel of subject-matter expert’s review the items in terms of content adequacy.  - These experts should be provided with construct definitions and instructed to sort the item s according to these definitions, to determine whether their sort aligns with the scale developer’s conceptualisations. |
| Reliability [1,4] | - It is the amount of variance attributable to the true score of the latent construct and to the repeatability, stability or consistency of a tool.  - Test–retest reliability can assess the stability of a measure over time.  - Tool development and reliability do not provide evidence of a tool's effectivity or effect. |
| Validity [1,4] | - It refers to whether a tool is measuring what it purports to.  - There are several different types of validity: content validity, convergent, discriminant validity, and predictive validity.  - Testing the construct (external validity) consists of the confirmation of factor structure in an independent data set, using exploratory and confirmatory methods. |
| Test [2,3,5] | - The final questionnaire was administered to an independent sample.  - In the validity and test stages the psychometric characteristics of the tool must be calculated. |

**References**

1. Kumar A. Review of the Steps for Development of Quantitative Research Tools. *J Adv Pract Nurs* 2015;**1**:103.

2. Altman DG, Bland JM. Diagnostic tests 2: Predictive values. *BMJ* 1994;**309**:102.

3. Altman DG, Bland JM. Diagnostic tests 1: Sensitivity and specificity. *BMJ* 1994;**308**:1552.

4. Parameaswari PJ. Tool development in health care research. *J Med Allied Sci* 2013;**3**:3–7.

5. Gjersing L, Caplehorn JR, Clausen T. Cross-cultural adaptation of research instruments: Language, setting, time and statistical considerations. *BMC Med Res Methodol* 2010;**10**:13.
